# Determinants of poor glycaemic control and proteinuria in patients with type 2 diabetes: a retrospective analysis of general practice records in Ireland

**DOI:** 10.1186/s12875-023-02252-w

**Published:** 2024-01-10

**Authors:** Maria Sullivan, Raymond O’Connor, Ailish Hannigan

**Affiliations:** 1 School of Medicine, University of Limerick, Limerick, Ireland; 2 Health Research Institute, University of Limerick, Limerick, Ireland

**Keywords:** Type 2 diabetes, Glycaemic control, General practice

## Abstract

**Background:**

Analysis of general practice records can address the information gap on the epidemiology of type 2 diabetes (T2DM) in Ireland, informing practice and the development of interventions in primary care. The aim of this study was to identify patients with poor glycaemic control, risk factors for complications and evidence of end organ damage in a large multi-practice study and to profile their characteristics.

**Methods:**

Patients with T2DM were identified using disease coding in Health One practice management software in 41 general practices. Patients’ demographics and clinical data were extracted. Rates of poor glycaemic control (glycated haemoglobin > 58 mmol/mol) and albumin creatinine ratio > 3 mg/mmol were calculated. A multilevel logistic regression analysis using both patient and practice variables was conducted.

**Results:**

Data was collected from 3188 patients of whom 29% (95% CI 28 to 31%) had poor glycaemic control, which was associated with younger age, higher BMI and higher total cholesterol. Only 42% of patients (n = 1332) had albumin creatinine ratio measured with 42% (95% CI 40 to 45%) of these having values > 3 mg/mmol. Older age groups, men, those with hypertension, eGFR < 60 ml/min/1.73m^2^ and poor glycaemic control were most associated with higher values of albumin creatinine ratio.

**Conclusions:**

Analysing this large multi-practice dataset gives important information on the prevalence and characteristics of diabetic patients who are most at risk of poor outcomes. It highlights that recording of some data could be improved.

## Background

Type 2 diabetes (T2DM) is a complex and chronic disease which if not controlled can lead to acute life-altering complications [1]. Its global prevalence is expected to increase from 537 million in 2021 to 643 million in 2040 [2]. The detrimental health effects of T2DM complications on the patient, and the cost of disease management to the health care system, have made the question of how best to manage T2DM a central clinical and health planning topic [1]. T2DM leads to serious macrovascular and microvascular complications such as coronary artery disease, cerebrovascular accidents, diabetic retinopathy which can lead to blindness, diabetic nephropathy which can lead to renal failure and peripheral neuropathy leading to non-traumatic lower limb amputations [2, 3]. To prevent disease and complications of T2DM, understanding the physiological and biochemical marker changes is important [4]. Proteinuria is strongly associated with diabetic kidney disease and diabetic retinopathy [3, 5]. Proteinuria not only functions as a clinical indicator for diabetic kidney disease, but it also plays a vital role in disease progression [6, 7]. Diabetic kidney disease accounts for approximately 40% of renal failure [8, 9].

Poor glycaemic control is a major risk factor for microvascular complications and the risk of developing those complications is directly related to the magnitude of the glycated haemoglobin (HbA1c) [10–13]. Obesity, hypertension, dyslipidaemia and tobacco smoking have all been associated with increased risk of complications and these parameters all have the potential to be treated and controlled in a primary care setting [14, 15]. Regular screening can also take place to identify complications early [2].

In the Republic of Ireland, it is estimated that there are approximately 207,000 people with T2DM [16, 17]. In 2015 the ‘cycle of care’ program was introduced which allowed patients with a medical card or GP visit card two free general practice consultations per year to manage and prevent their diabetes complications. Incentivising these visits can be an effective way to improve population health [18] and analysis of these records can address the information gap on the epidemiology of T2DM in Ireland where no national register for diabetes exists. The aim of this study was to identify patients with poor glycaemic control, risk factors for complications and evidence of end organ damage in a large multi-practice study and to profile their characteristics. We will compare rates across practices and account for both practice and patient level characteristics to understand poor glycaemic control and proteinuria. The results can be used to inform clinical practice, as well as the development of interventions for patients with T2DM in primary care.

## Methods

### Participants

This is a secondary analysis of data extracted to study the implementation of the ‘cycle of care’ and how this affected management systems of those with T2DM in Ireland between 2014 and 2017 [18]. At the time of the data collection, 51% of patients with T2DM were registered with the ‘cycle of care’ program. This accounted for 103,800 patients in Ireland [16–18]. Invitations to take part were sent to practices using a discussion forum for Health One clinical software. This software is used in approximately 400 practices in Ireland, and 50% of these use the discussion forum. Participating practices provided data on patients with T2DM. Data on eligible patients was extracted from the electronic medical record system of participating practices using secure customised software [18]. Using the International Classification of Diseases (ICD-10) and International Classification of Primary Care coding system, patients with T2DM were identified from general practice records using a unique code [18].

### Data collected

This analysis focuses on the data collected in 2017, 12 months after the ‘cycle of care’ was introduced. The data collected included:

1. Demographic data on patients with T2DM. This included the age and sex of patients and their risk factors such as Body Mass Index (BMI) and smoking status.
2. Clinical data – Targets of HbA1c ≤ 58 mmol/mol, total cholesterol < 5 mmol/litre, blood pressure ≤ 140/80 mmHg, Albumin Creatinine ratio (ACR) < 3 were used as advised by National Institute for Health Care Excellence (NICE) and the American Diabetes Association [19–21]. The lipid parameters LDL < 3.0 mmol/L, HDL > 1.0 mmol/L and triglycerides < 1.8 mmol/L were taken from NICE guidelines and the European Society of Cardiology (ESC) and European Atherosclerosis Society (EAS) [21–23]. Where multiple values existed for some clinical parameters, a mean value was used to summarise the parameter for an individual patient.

### Data analysis

Data from each practice was anonymised and amalgamated into a single master file. Descriptive statistics were used to characterise participating practices, patient demographics, and clinical data. Proportions of patients with poor glycaemic control (Hba1c > 58 mmol/mol) and ACR > 3 mg/mmol are reported with 95% confidence intervals. Descriptive statistics were used to compare the characteristics of patients with poor glycaemic control or not and patients with ACR > 3 mg/mmol or not. The chi-squared test was used to test the association between categorical variables and group. The Mann-Whitney test compared medians across groups. The independent samples t-test compared means across groups. A multilevel binary logistic regression model was fitted to explain poor glycaemic control (yes, no), adjusting for age group, sex, BMI category, smoking status, eGFR category, systolic blood pressure and cholesterol and accounting for the structure of patients clustered within practices. A multilevel binary logistic regression model was fitted to explain ACR > 3 mg/mmol (yes, no), adjusting for age group, sex, BMI category, smoking status, eGFR category, systolic blood pressure, cholesterol and glycaemic control and accounting for the structure of patients clustered within practices. Adjusted odds ratios (aOR) with 95% confidence intervals (CI) are reported. Goodness of fit was assessed using the Hosmer-Lemeshow chi-squared test. IBM SPSS Statistics for Windows (version 25) was used to carry out the analysis and a 5% level of significance used for all tests.

## Results

**Fig. 1:**
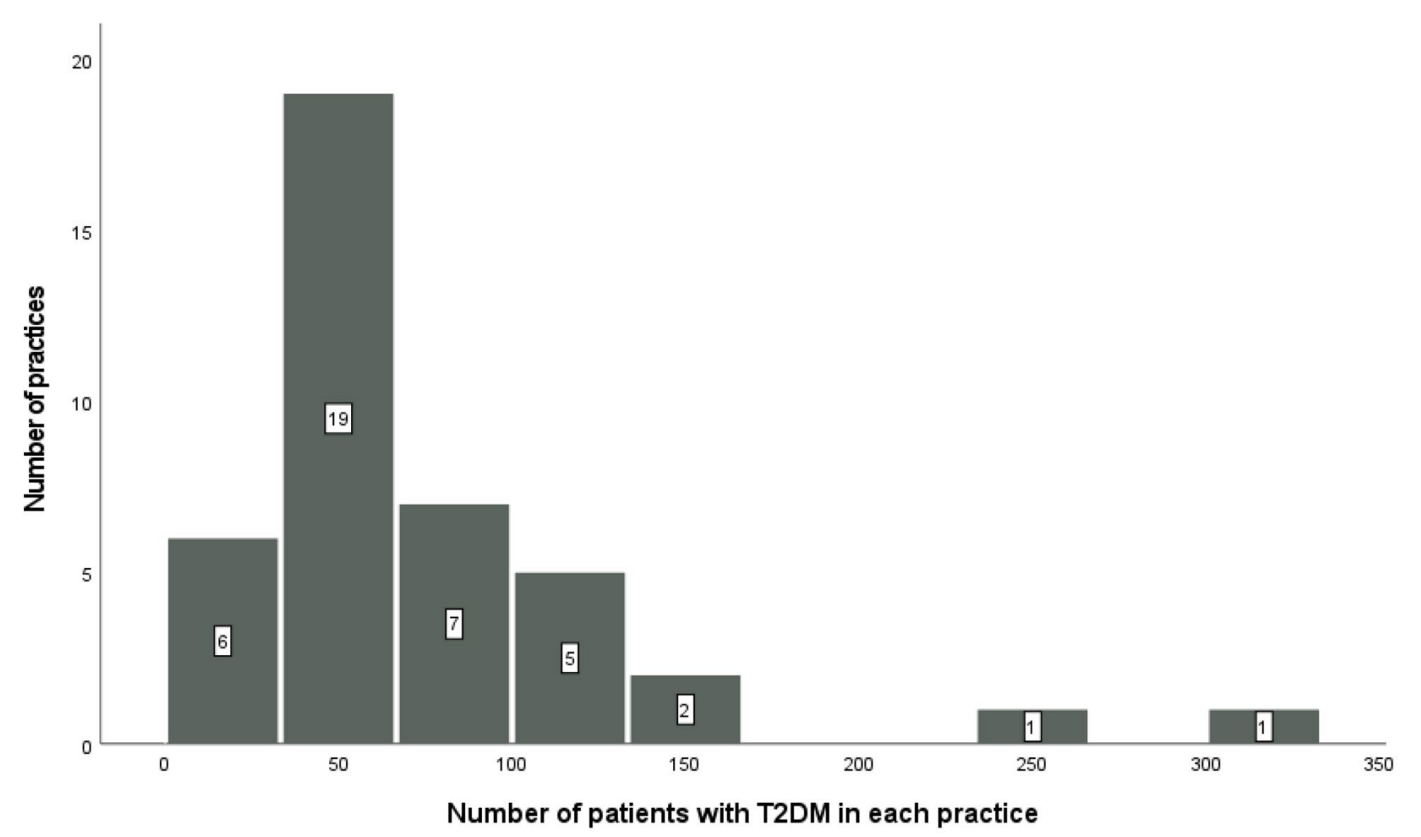
Number of patients with T2DM in each practice (n = 41 practices)

Of 250 practices in the discussion forum, 41 practices participated (response rate 16.4%), yielding data from 3188 patients with T2DM. The number of patients with T2DM from each practice varied from 16 to 333 with a median of 57 patients (Fig. 1).

Table 1 summarises the characteristics of the 3188 patients. The majority were male (n = 1780, 55.8%) and aged 70 years or older (54.8%). The majority (n = 2639, 82.8%) were overweight or obese. 17.4% were smokers.

**Table 1:** Characteristics of patients (n = 3188)

Patient characteristics	n (%)	Median (Q1, Q2)
**Age**		71 (61, 78)
**Age group (years)**		
< 50 50–59 60–69 70–79 ≥80	277 (8.7%) 456 (14.3%) 709 (22.2%) 1175 (36.9%) 571 (17.9%)	
**Gender**		
Male Female	1780 (55.8%) 1408 (44.2%)	
**BMI (kg/m** ^**2**^ **)**		30 (27, 34)
<25 25-29.9 30-34.9 ≥35 Missing	428 (13.4%) 1033 (32.4%) 932 (29.2%) 674 (21.1%) 121 (3.8%)	
**Smoking status**		
Yes No Missing	555 (17.4%) 2593 (81.3%) 40 (1.3%)	

**Fig. 2:**
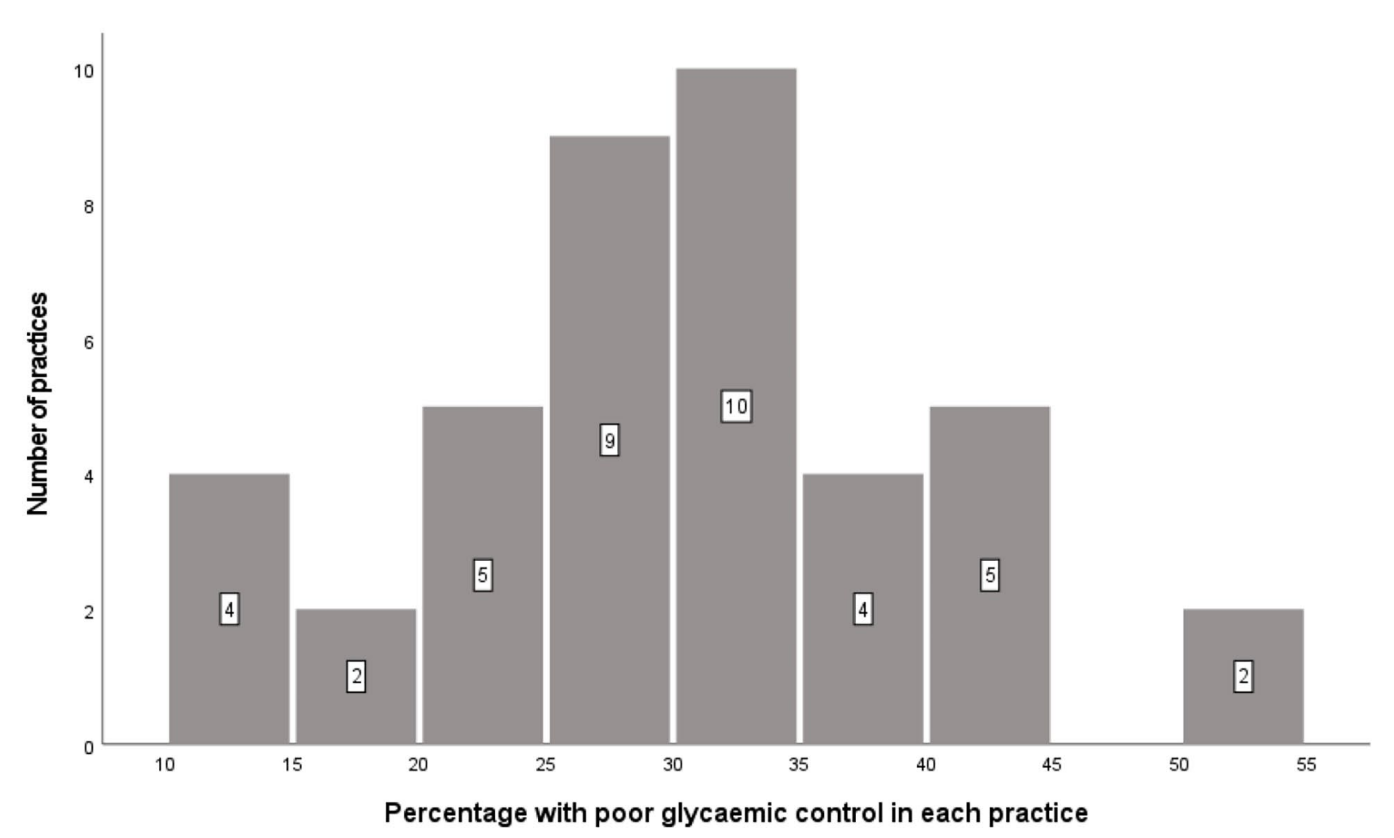
Percentage with poor glycaemic control in each practice (n = 41 practices)

Using a cut-off of HbA1c > 58 mmol/mol, 29.3% (95% CI 27.7–30.9%) of patients had poor glycaemic control. The percentage with poor glycaemic control in each practice varied from 10.2 to 52.8% (Fig. 2).

Table 2 summarises the characteristics of the patients with HbA1c ≤ 58 and > 58 mmol/mol.

**Table 2:** Characteristics of those with and without poor glycaemic control (n = 3097)^1^

Variable	HbA1c ≤ 58 (n = 2191)	HbA1c > 58 (n = 906)	*P*-Value
Patient Characteristics			
**Age (years)**			
Under 50 50–59 60–69 70–79 ≥80	153 (7.0%) 263 (12.0%) 489 (22.3%) 861(39.3%) 425(19.4%)	110 (12.1%) 175(19.3%) 198 (21.9%) 286(31.6%) 137(15.1%)	< 0.001
Median age (Q1, Q2)	72 (63, 78)	68 (57, 76)	< 0.001
**Gender**			
Male Female	1210 (55.2%) 981 (44.8%)	521 (57.5%) 385 (42.5%)	0.245
**BMI (kg/m** ^**2**^ **)**			
<25 25-29.9 30-34.9 ≥35	321 (15.0%) 745 (34.8%) 639 (29.9%) 434 (20.3%)	98 (11.0%) 282 (31.6%) 282 (31.6%) 230 (25.8%)	< 0.001
**Smoker**			
Yes No	364 (16.6%) 1827 (83.4%)	179 (19.8%) 727 (80.2%)	0.036
**Clinical/Lab Variables**			
**Total cholesterol (mmol/L)**			
≤5 >5	1794(82.3%) 387 (17.7%)	698(78.0%) 197(22.0%)	0.006
**LDL (mmol/L)**			
<3 ≥3 Missing	1723 (78.6%) 399 (18.2%) 69 (3.1%)	681 (75.2%) 176 (19.4%) 49 (5.4%)	0.006
**eGFR (**ml/min/1.73m^2^)			
≥60 <60 Missing	1341 (61.2%) 437 (19.9%) 413 (18.8%)	538 (59.4%) 179 (19.8%) 189 (20.9%)	0.428
**Systolic blood pressure (mmHg)**			
≤140 >140	1455 (67.3%) 708 (32.7%)	598 (67.1%) 293 (32.9%)	0.935

^1^ 91 with missing values for HbA1c

The median age was lower in the poorly controlled group at 68 years compared to 72 years for the controlled group (*p* < 0.001). There was an association between BMI and glycaemic control (*p* < 0.001) with a larger percentage of those in the poorly controlled group having a BMI of 35 kg/m^2^ or greater (25.8% compared to 20.3% in the controlled group). Rates of total cholesterol > 5 mmol/L were higher in the poorly controlled group (22% vs. 18%, *p* = 0.006). Rates of eGFR < 60 ml/min/1.73m^2^ and systolic blood pressure > 140 mmHg were similar in both groups (see Table 2).

In a multivariable logistic regression model accounting for all the variables in Table 2 and the clustering of patients within practices, younger patients had higher odds of poorer glycaemic control [aOR 2.44 (95% CI 1.68 to 3.53) for those aged less than 50 compared to those aged 80 years and older] (see Table 3). Those with a BMI of 35 kg/m^2^ or greater also had higher odds of poorer glycaemic control [aOR 1.40 (95% CI 1.04, 1.90) compared to those with a BMI < 25 kg/m^2^]. Those with a total cholesterol > 5 mmol/L also had higher odds of poorer glycaemic control [aOR 1.39 (95% CI 1.04, 1.87) compared to those with total cholesterol ≤ 5mmol/L] (see Table 3).

A subset of patients had ACR measured (n = 1332, 42% of patients). Patients with ACR measured were more likely to also have an eGFR recorded (0.8% missing data for eGFR compared to 35.3% for those without ACR). They were also more likely to have an eGFR < 60 ml/min/1.73m^2^ compared to those without ACR measured (24.8% compared to 15.6%).

Of the patients with ACR measured, 562 (42.2%, 95% CI 39.5 to 44.9%) had ACR values > 3 mg/mmol. Table 4 summarises the characteristics of the patients with ACR > 3 mg/mmol or not. Patients with ACR > 3 mg/mmol were older with a median age of 73 years compared to 70 for those with an ACR ≤ 3 (*p* < 0.001). They were also more likely to be male (64% vs. 58%, *p* = 0.023), have an eGFR < 60 ml/min/1.73m^2^, systolic blood pressure over 140mmHg and have poor glycaemic control (See Table 4).

**Table 3:** Binary logistic regression^1^ for poor glycaemic control (n = 2973)

Variable	Odds ratio (95% CI)	*P*-Value
Patient Characteristics		
**Age (years)**		
Under 50 50–59 60–69 70–79 ≥80	2.44 (1.68, 3.53) 2.18 (1.59, 2.99) 1.36 (1.02, 1.80) 1.09 (0.85, 1.41) Reference	< 0.001
**Gender**		
Male Female	Reference 0.89 (0.75, 1.06)	0.19
**BMI (kg/m** ^**2**^ **)**		
<25 25-29.9 30-34.9 ≥35	Reference 1.16 (0.88, 1.54) 1.30 (0.98, 1.73) 1.40 (1.04, 1.90)	0.11
**Smoker**		
Yes No	1.05 (0.83, 1.33) Reference	0.68
**Clinical/Lab Variables**		
**Total cholesterol (mmol/L)**		
≤5 >5	Reference 1.39 (1.04, 1.87)	0.03
**LDL (mmol/L)**		
<3 ≥3 Missing	Reference 0.78 (0.58, 1.06) 1.49 (0.92, 2.42)	0.04
**eGFR (ml/min/1.73m** ^**2**^ **)**		
<60 ≥60 Missing	Reference 0.80 (0.63, 1.00) 0.86 (0.65, 1.13)	0.14
**Systolic blood pressure (mmHg)**		
≤140 >140	Reference 1.08 (0.90, 1.30)	0.41

^1^ With random effect for general practices to account for clustering of patients within practices

**Table 4:** Characteristics of patients with ACR ≤ 3 and > 3 (n = 1332). ACR units are in mg/mmol

	ACR ≤ 3 (n = 770)	ACR > 3 (n = 562)	*P* value
Patient Characteristics			
**Age (years)**			
Under 50 50–59 60–69 70–79 ≥80	76 (9.9%)) 111 (14.4%) 197 (25.6%) 274 (35.6%) 112 (14.5%)	40 (7.1%) 58 (10.3%) 100 (17.8%) 231 (41.1%) 133 (23.7%)	< 0.001
**Median age (Q1, Q2)**	70 (60, 76)	73 (65, 79)	< 0.001
**Gender**			
Male Female	447 (58.1%) 323 (41.9%)	361 (64.2%) 201 (35.8%)	0.023
**BMI (kg/m** ^**2**^ **)**			
<25 25-29.9 30-34.9 >=35	87 (11.4%) 275 (36.0%) 233 (30.5%) 168 (22.0%)	70 (12.6%) 200 (36.0%) 172 (30.9%) 114 (20.5%)	0.863
**Smoker**			
Yes No	106 (13.8%) 664 (86.2%)	80 (14.2%) 482 (85.8%)	0.807
**Clinical/Lab values**			
**Total Cholesterol (mmol/L)**			
≤5 >5	634 (82.3%) 136 (17.7%)	472 (84.0%) 90 (16.0%)	0.429
**LDL (mmol/L)**			
<3 ≥3	608 (81.3%) 140 (18.7%)	456 (83.2%) 92 (16.8%)	0.37
**Systolic blood pressure (mmHg)**			
≤140 >140	525 (69.3%) 233 (30.7%)	324 (58.4%) 231 (41.6%)	< 0.001
**eGFR (ml/min/1.73m** ^**2**^ **)**			
≥60 <60	613 (80.3%) 150 (19.7%)	378 (67.7%) 180 (32.3%)	< 0.001
**HbA1c (mmol/mol)**			
≤58 >58	583 (75.8%) 186(24.2%)	377 (67.1%) 185 (32.9%)	< 0.001
**Median HbA1c (Q1, Q2)**	50.5 (45, 58)	53 (46.5, 61)	0.01

In a multivariable logistic regression model accounting for all the variables in Table 4 and the clustering of patients within practices, older patients had higher odds of ACR > 3 mg/mmol [aOR 2.26 (95% CI 1.25 to 4.09) for those aged 80 years or older compared to those aged under 50] (see Table 5). Male patients also had higher odds of ACR > 3 mg/mmol compared to female patients [aOR 1.34 (95% CI 1.03, 1.74)]. Those with poorer glycaemic control also had higher odds of ACR > 3 mg/mmol compared to those with HbA1c < 58 mmol/mol [aOR 1.76 (95% CI 1.33, 2.34)] (see Table 5). Patients with hypertension and patients with eGFR < 60 ml/min/1.73m^2^ also had higher odds of ACR > 3 mg/mmol (see Table 5).

**Table 5:** Binary logistic regression^1^ for ACR > 3 mg/mmol (n = 1288)

Variable	Odds ratio (95% CI)	*P*-Value
Patient Characteristics		
**Age (years)**		
Under 50 50–59 60–69 70–79 ≥80	Reference 0.90 (0.50, 1.61) 0.80 (0.47, 1.39) 1.41 (0.83, 2.39) 2.26 (1.25, 4.09)	< 0.001
**Gender**		
Male Female	1.34 (1.03, 1.74) Reference	0.03
**BMI (kg/m** ^**2**^ **)**		
<25 25-29.9 30-34.9 ≥35	Reference 0.91 (0.60, 1.38) 1.11 (0.72, 1.70) 1.12 (0.70, 1.78)	0.55
**Smoker**		
Yes No	1.10 (0.74, 1.63) Reference	0.65
**Clinical/Lab Variables**		
**Total cholesterol (mmol/L)**		
≤5 >5	Reference 0.89 (0.53, 1.50)	0.67
**LDL (mmol/L)**		
<3 ≥3 Missing	Reference 1.00 (0.60, 1.67) 1.86 (0.62, 5.60)	0.53
**eGFR (ml/min/1.73m** ^**2**^ **)**		
<60 ≥60	1.89 (1.39, 2.56) Reference	< 0.001
**Systolic blood pressure (mmHg)**		
≤140 >140	Reference 1.75 (1.34, 2.29)	< 0.001
**HbA1c (mmol/mol)**		
≤ 58 > 58	Reference 1.76 (1.33, 2.34)	< 0.001

^1^ With random effect for general practices to account for clustering of patients within practices

## Discussion

Our study of 3188 patients with T2DM cared for by GPs showed that 29% (95% CI 28 to 31%) had poor glycaemic control, which was associated with younger age, higher BMI and higher total cholesterol. This prevalence rate is comparable to previous data collected in Ireland. The audit report of the Health Service Executive Midlands structured care program in 2010 showed that 25.6% of patients were in this high-risk category (> 58mmol/mol) [24]. In the UK national diabetes audit conducted by the NHS on 3,136,070 patients in primary care, 33.2% had poorly controlled HbA1c [20]. Only a subset of patients in our study (n = 1332, 42%) had ACR measured with 42% (95% CI 40 to 45%) of these having values > 3 mg/mmol. Older age groups, men, those diagnosed with hypertension, eGFR < 60 ml/min/1.73m^2^ and with poor glycaemic control were most associated with higher ACR values.

In this study, the median age of those with poor glycaemic control was four years younger (median 68 years) than the controlled group (median 72 years). The prevalence of T2DM in those under 50 is dramatically increasing. Almost one in 10 (9%) of the patients in this study are aged under 50 and the youngest patient in this sample is 16. The prevalence of young onset T2DM is not well documented in Ireland. Diabetes in young people has a longer disease exposure and increased risk for complications. Younger patients have been shown to have a higher prevalence of family history of diabetes, hypertension, and worse glycaemic control than later-onset patients [25–28]. The younger patients in this study had a higher risk of poor glycaemic control compared to the oldest patients. The younger onset T2DM has proven to have a different more aggressive disease phenotype which may lead to poor control [25]. This is turn would lead to premature development of complications, adverse effects on quality of life and unfavourable effects on long-term outcomes, raising the possibility of a future public health catastrophe [25, 28].

Men are more likely to have T2DM (56% of this sample) and men were also more likely to have higher ACR (> 3 mg/mmol). Men’s poorer glycaemic control and subsequent complications have been well documented, and research has shown that men with T2DM are more likely to have ST Elevation Myocardial Infarctions (STEMIs) and non-STEMIs (NSTEMIs) [29] and ischemic stroke [30]. Men are more likely to be diagnosed at a lower BMI but they may still have higher visceral fat and this should be a consideration when screening patients [31].

The majority (83%) of patients in this study were overweight or obese. The median body mass index of patients was 30 kg/m^2^ with over half of the patients categorised as obese. This result is similar to the audit of the diabetes structured care program in 2010 [24]. Those in the highest BMI category (BMI of > 35 kg/m^2^) in our study were more likely to have poor glycaemic control. Ireland has one of the highest levels of obesity in Europe, with 60% of adults and over 20% of children and young people overweight and obese. The prevalence of obesity among children, adolescents, and young adults with T2DM is much greater than in older adults with T2DM and this is something that general practitioners can be aware of with their younger patients [25]. BMI levels were similar in this study between groups with high and low ACR. Research has so far not established a relationship with obesity and ACR [32].

Despite growing evidence of the impact of smoking on diabetes complications, people with T2DM still smoke. 17.4% of this sample size were smokers which was lower than the national average of 21% and a decrease from the national diabetes audit which was 20.4% [24]. The smokers were more likely to have poorer glycaemic control and this is in line with current research [4, 33]. Smoking and diabetes can act synergistically on morbidity and mortality [34–36].

Diabetic kidney disease is the current cause of half of chronic kidney disease (CKD) cases across regions of the world [37]. Those with optimal eGFR > 60 ml/min/1.73m^2^ were more likely to have better glycaemic control. A subset of patients had ACR measured. This subset were more likely to also have eGFR measured and have an eGFR < 60 ml/min/1.73m^2^. They may represent, therefore, a subset of patients perceived to be at higher risk of complications by their GP. Full evaluation of renal function necessitates the simultaneous measurement of ACR. Of those with ACR measured, 42% had an ACR of greater than 3 mg/mmol which is comparable to the 39% of patients in the diabetes structured care program audit in 2010 [24]. The percentage of patients with evidence of albuminuria varies globally from 15 to 45% [38–40]. The median age in the high ACR group was three years older than the lower ACR group. The physiological decline of renal function with age may have also played a role since senescence is associated with a gradual decline of kidney function [41, 42]. The American Diabetes Association and Irish College of General Practitioners have recommended strict treatment of hypertension in the setting of T2DM [24, 43]. Those with uncontrolled hypertension were more likely to have increased ACR. Hypertension‘s effect on the kidney is well established and hypertension is one of the most common causes of chronic kidney disease. Those with low eGFR in this study were more likely to have an elevated ACR. Albuminuria and hypertension are independently associated with an increased risk of all-cause mortality [44].

We identified practice level variation in the percentage of patients with poor glycaemic control, similar to the findings of Heald et al. on data from the UK National Diabetes Audit [45]. Further research is required to understand why these practice differences occur and the causes of variation in processes of care in Ireland. Previous research in Ireland has shown that the incentivising ‘cycle of care’ payment scheme for these patients improved the amount of data recorded on the processes of care and has also improved the outcomes for parameters such as cholesterol and blood pressure but not HbA1c [18]. The HSE structured chronic disease management programme has been extended to all patients with T2DM over the age of 18 which will allow for comprehensive general practice and national data on T2DM. The usefulness of this data may benefit from recording additional data such as ethnicity, highest level of education, comorbidities, the length of time since diagnosis, mental health status, activity levels, waist circumference, family history, levels of vitamin D and what pharmacological assistance they are receiving [25, 46, 47].

### Strengths and limitations

This study is currently the largest sample (n = 3188) of T2DM patients that has been analysed in primary care in Ireland since structured diabetes care was introduced in 2016. This study was a large multi-practice study which included patient demographics, laboratory data, and practice data. Its most comparable data was from the Midlands diabetes audit collected in 2010 which collected similar data on 989 patients. Parameters such as rates of glycaemic control, obesity levels and kidney function were similar which highlights the difficulty in improving these parameters in the population.

The limitations in this data set were that some data, namely ACR, was poorly recorded in practices (58% of patients with missing data). The Irish College of General Practitioners diabetes guidelines advise that ACR is recorded at diagnosis of T2DM and annually thereafter for all patients [48]. Microvascular complications have been shown to increase with duration of diabetes [49] and all patients in this dataset were a minimum of three years from diagnosis so we cannot generalise to newly diagnosed patients. Only patients eligible for free visits to their general practitioner were included and we do not have data on fee-paying patients.

## Conclusions

Younger patients were more likely to have poorer glycaemic control. The more aggressive T2DM phenotype should be a consideration for general practitioners in the care of younger patients. Patients with high BMIs were also more likely to have poor glycaemic and lipid control and metabolic syndrome should be considered for these patients. These characteristics identified could be used by general practitioners to focus care on those most at risk for complications and mortality from T2DM. This study also reported variation between practices, and this is something that requires further research. For general practitioners, this study may also highlight the importance of recording data on processes of care, improving the knowledge available on T2DM patients in Ireland and promoting the delivery of safe and effective care. Further research should repeat these measurements in all patients now registered in the chronic disease management program and conduct longer term follow-up on these patients.

## Data Availability

The individual level data is not publicly available. The minimal dataset used is available from the corresponding author on reasonable request.

## Abbreviations

T2DM: Type 2 diabetes
HbA1c: Glycosylated haemoglobin
ACR: Albumin creatinine ratio
eGFR: Estimated glomerular filtration rate
